# Magnetic resonance imaging of cerebrospinal fluid outflow after low-rate lateral ventricle infusion in mice

**DOI:** 10.1172/jci.insight.150881

**Published:** 2022-02-08

**Authors:** Yann Decker, Jonas Krämer, Li Xin, Andreas Müller, Anja Scheller, Klaus Fassbender, Steven T. Proulx

**Affiliations:** 1 Department of Neurology, Saarland University Medical Center, Homburg, Germany.; 2 Theodor Kocher Institute, University of Bern, Bern, Switzerland.; 3 Clinic for Diagnostic and Interventional Radiology and; 4 Molecular Physiology, Center for Integrative Physiology and Molecular Medicine, University of Saarland, Homburg, Germany.

**Keywords:** Neuroscience, Vascular Biology, Lymph, Neuroimaging

## Abstract

The anatomical routes for the clearance of cerebrospinal fluid (CSF) remain incompletely understood. However, recent evidence has given strong support for routes leading to lymphatic vessels. A current debate centers upon the routes through which CSF can access lymphatics, with evidence emerging for either direct routes to meningeal lymphatics or along cranial nerves to reach lymphatics outside the skull. Here, a method was established to infuse contrast agent into the ventricles using indwelling cannulae during imaging of mice at 2 and 12 months of age by magnetic resonance imaging. As expected, a substantial decline in overall CSF turnover was found with aging. Quantifications demonstrated that the bulk of the contrast agent flowed from the ventricles to the subarachnoid space in the basal cisterns. Comparatively little contrast agent signal was found at the dorsal aspect of the skull. The imaging dynamics from the 2 cohorts revealed that the contrast agent was cleared from the cranium through the cribriform plate to the nasopharyngeal lymphatics. On decalcified sections, we confirmed that fluorescently labeled ovalbumin drained through the cribriform plate and could be found within lymphatics surrounding the nasopharynx. In conclusion, routes leading to nasopharyngeal lymphatics appear to be a major efflux pathway for cranial CSF.

## Graphical abstract

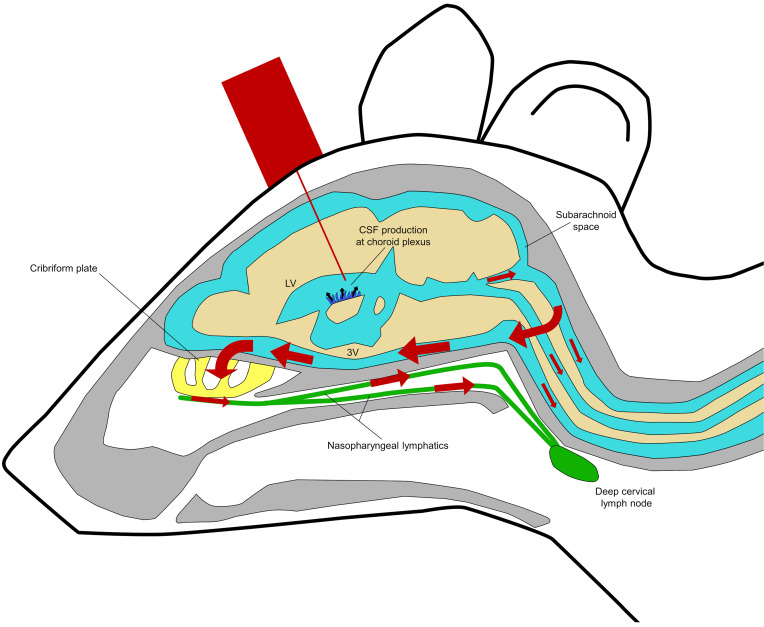

## Introduction

Cerebrospinal fluid (CSF) is produced within the ventricles by the choroid plexuses and circulates within the subarachnoid spaces around the brain and spinal cord. Historically, it was concluded that CSF leaves the central nervous system (CNS) via direct pathways through outcroppings of arachnoid tissue into the venous sinuses of the dura mater (1, 2). However, in recent decades it has become commonly accepted that lymphatic vessels play a significant role in the process of CSF drainage (3–6). Recent studies in rodents have demonstrated that lymphatic vessels appear to be the exclusive clearance route for tracers injected into the CSF, even for low–molecular weight solutes (7, 8). An active area of research has focused upon the anatomical routes of outflow that CSF takes to access the lymphatic vessels. Support exists for access of CSF to lymphatic vessels that have been recently rediscovered in the dura mater (9–11) and for efflux along cranial or spinal nerves to reach extracranial lymphatics (7, 12–15). Of the perineural routes, evidence in many different species exists for routes along olfactory nerves through the cribriform plate (5, 10, 16–20).

Imaging techniques have long been utilized to assess CSF outflow of tracers (6). Traditionally, these techniques have employed x-ray or scintigraphic measurements (21, 22). Recently, 2-photon and near-infrared fluorescence techniques (7–9, 13, 14, 23) have been developed to allow sensitive in vivo readouts to allow sensitive in vivo readouts within the CNS (imaging through skull or through spine), at specific efflux pathways (imaging through vomeronasal bones into the nasal region or imaging of lymphatic vessels draining to the superficial cervical or mandibular lymph nodes), or to the systemic blood from multiple efflux pathways. However, these methods can only allow assessments at one particular region at a relatively superficial level. On the other hand, contrast-enhanced magnetic resonance imaging (CE-MRI) techniques have the advantage of 3D imaging of the entire cranial or spinal regions in the context of the surrounding soft tissue anatomy, with sufficient temporal resolution to track the dynamics of contrast agent outflow, and have a more immediate translational potential to the clinic (8, 11–13, 15, 24).

One valid criticism of tracer injection studies is that they may inherently introduce artifacts because of relatively large acute volumes introduced into the CSF. In the mouse, with an estimated CSF volume of 35 μL (2), injections of 5 to 10 μL made within a short period likely introduce elevated pressure conditions that will affect the dynamics and routes of outflow (8, 25). Our group has attempted to address this issue by establishment of an indwelling cannula into the lateral ventricle of mice that allows slow infusion during imaging acquisition (13) at rates below the published rates for CSF production (2, 26). We have utilized this cannula system to allow MRI measurements of spinal CSF distribution and sacral outflow during the infusion of contrast agent (13). Similar cisterna magna infusion setups have also recently been employed by other groups to examine cranial CSF distribution (12, 15).

Alterations in CSF circulation may have significant effects on the pathogenesis of neurological conditions associated with the aging process, including dementia and stroke. Many research groups have now demonstrated that a slower overall efflux of CSF occurs during the aging process (7, 11, 14). Thus, we have utilized this expected difference in CSF turnover dynamics between younger (2–3 months old) and older (12 months old) mice to aid in validation of MRI quantifications. Second, we have investigated potential efflux routes by analyzing the contrast agent signal dynamics at several locations within the CNS and the cervical lymph nodes. We hypothesized that differences in signal dynamics quantified between the 2 age groups would be apparent only at the major site(s) of efflux.

## Results

### Tracer infused into the lateral ventricle follows a continuous outflow route from the nasal cavity to the draining cervical lymph nodes.

**Figure 1:**
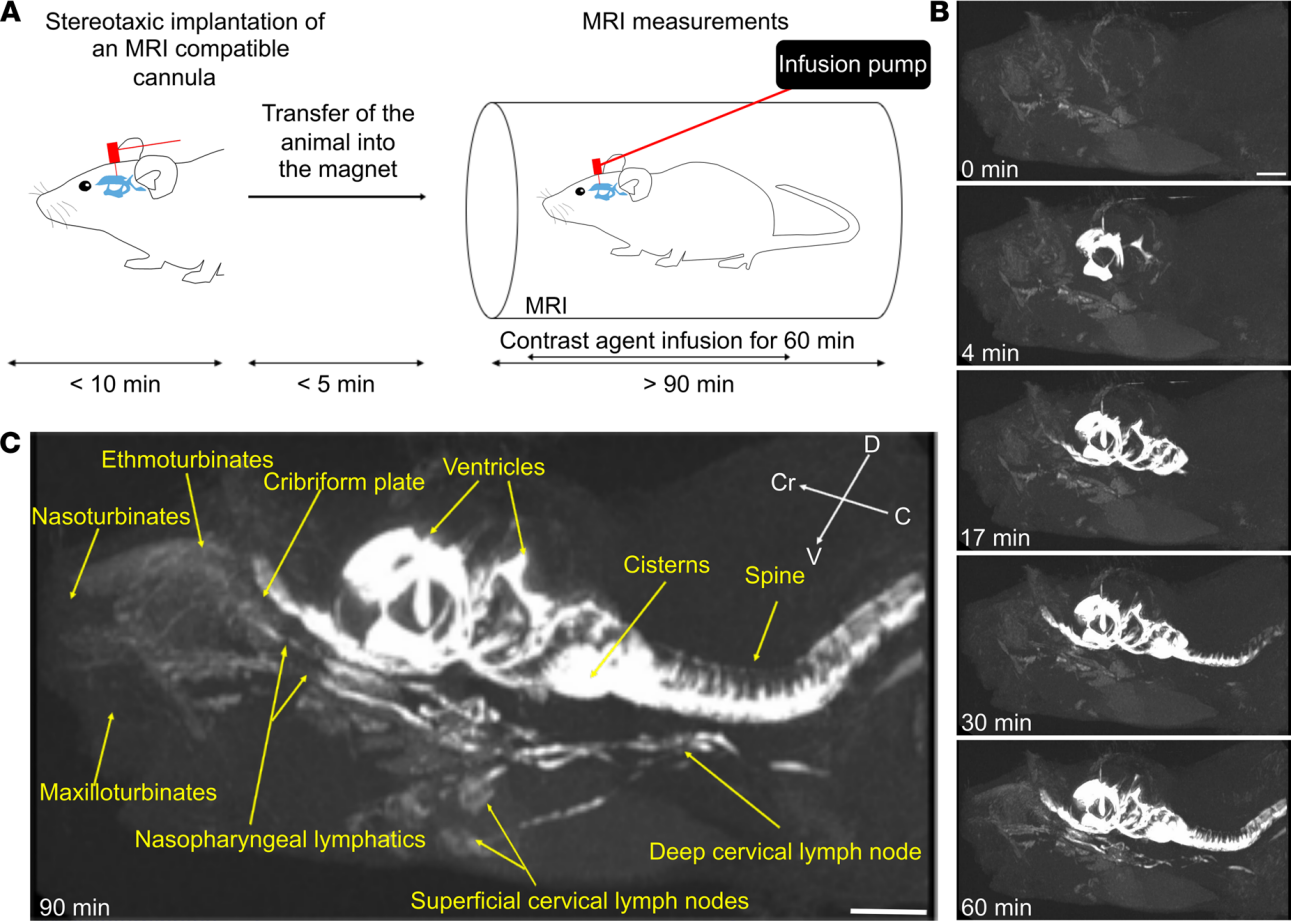
Dynamic CE-MRI shows continuous efflux of contrast agent from the nasal region through lymphatic vessels to cervical lymph nodes following low-rate ventricular infusion. (**A**) Schematic representation of the experimental setup. MRI-compatible cannulae were stereotactically implanted into the ventricle of 2- to 3-month-old C57BL/6J mice anesthetized with ketamine/xylazine. The animals were then transferred into a horizontal-bore 9.4 T MRI. Polyethylene tubing containing the contrast agent (GadoSpin D solution from nanoPET Pharma GmbH at 25 mM Gd) was attached connecting the cannula and the infusion pump. Before contrast agent infusion, T1-weighted (3D time-of-flight gradient recalled echo sequence) MRI measurements were started and followed by intraventricular low-rate infusion (0.1 μL/min) of the contrast agent while MRI acquisitions continued. (**B**) Representative signal dynamics using maximum-intensity projections visualizing the entire head-neck region. Following the beginning of contrast agent infusion, enhancement of the signal intensity in the ventricle is detectable at 4 minutes, in the nasal cavity at 17 minutes, and in the neck lymph nodes at 30 and 60 minutes. (**C**) Visualization of the spread of contrast agent after 90 minutes demonstrating continuous signal enhancement from the cribriform plate to the nasopharyngeal lymphatic vessels to cervical lymph nodes. Data are representative of *n* = 7 mice and 3 independent experiments. Scale bars: 3 mm.

Since CSF is principally produced by the choroid plexuses that are located in the ventricles, we decided to analyze the flow of tracer following an intraventricular low-rate infusion of a 17 kDa dendritic gadolinium-based contrast agent, GadoSpin D (Figure 1A). We chose GadoSpin D rather than a low–molecular weight contrast agent to limit potential diffusion into the brain parenchyma from the ventricular injection site. After stereotactic implantation of an indwelling cannula in the right lateral ventricle, mice were positioned in a prone position on a horizontal MRI platform. A polyethylene line filled with contrast agent was subsequently connected at one end to the cannula and at the other end to an infusion pump. After a baseline precontrast scan, an infusion of the contrast agent was started at a rate of 0.1 μL/min for 60 minutes, and a series of CE-MRI images were collected over the course of 90 minutes. This experimental setup allowed us to acquire images of the animal throughout the infusion of a macromolecular contrast agent at rates not excessive beyond physiological levels.

We first determined the general pattern of the spread of GadoSpin D by generating maximum-intensity projection images at each time point to allow a 3D dynamic visualization of the contrast agent (Supplemental Video 1; supplemental material available online with this article; https://doi.org/10.1172/jci.insight.150881DS1). An initial filling of the ventricular system was apparent, spreading ventrally to the basal cisterns and under the olfactory bulbs within the first 15 minutes after the start of the infusion. With increasing time, the signal intensity of the tracers was progressively detectable in the nasal cavity, in the nasopharyngeal region, and in the deep and superficial lymph nodes (Figure 1B). Moreover, as observed in Figure 1C and Supplemental Video 1, it was possible to identify a continuous anatomic route along the nasopharynx that emerged from the nasal cavity and connected to the deep cervical lymph nodes. Connections also were apparent from the deep cervical lymphatics to the superficial cervical (or mandibular) lymph nodes, as previously shown using near-infrared imaging (7).

### Reduced tracer clearance from the ventricles and slower efflux to draining lymph nodes in older mice.

**Figure 2:**
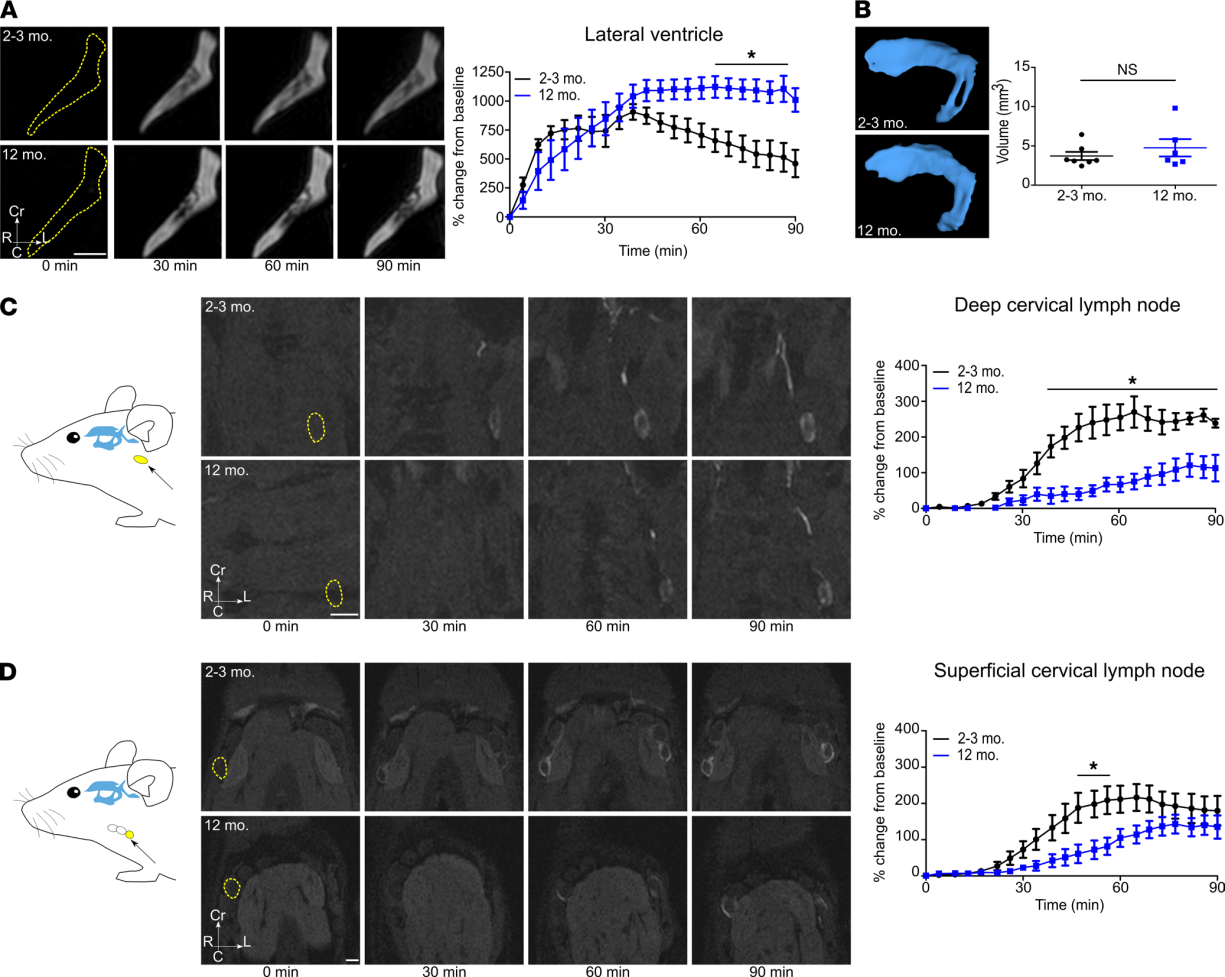
Clearance from ventricles and efflux to lymph nodes are reduced in 12-month-old mice. Visualization of tracer spread after low-rate intraventricular infusion (0.1 μL/min) of a GadoSpin D solution at 25 mM Gd; data acquired with a series of T1-weighted MRI measurements (3D time-of-flight gradient recalled echo sequence). (**A**) Signal dynamics of GadoSpin D contrast agent showing clearance from the contralateral ventricles in the horizontal plane in young (2–3 months) and 12-month-old mice. (**B**) Representative images of 3D reconstruction of the contralateral ventricles of young and 12-month-old mice. Ventricle volumes of young and 12-month-old mice were compared with 2-tailed Student’s *t* test. (**C** and **D**) Signal dynamics in the horizontal plane of GadoSpin D tracer efflux to deep and superficial cervical lymph nodes in young and 12-month-old mice. ROIs shown in yellow. Data are expressed as mean ± SEM of 2- to 3-month-old mice (*n* = 7) vs. 12-month-old mice (*n* = 6) and are representative of 3 independent experiments. *P < 0.05 (2-way ANOVA followed by Bonferroni’s post hoc test). Scale bars: 1 mm.

It has recently been demonstrated that CSF production and drainage are both reduced in aged mice, suggesting that the overall CSF turnover is slower with aging (7, 14, 27). During and immediately after the low-rate intraventricular infusion, we investigated the clearance of GadoSpin D from the ventricular system in 2 groups of mice of either 2–3 months (*n* = 7) or 12 months of age (*n* = 6). While the low-rate infusion of contrast agent took place during the first 60 minutes, we observed that the signal intensity in the contralateral ventricles reached a peak at around 40 minutes and then progressively decreased in both age groups (Figure 2A and Supplemental Figure 1A). At later time points, we could observe significantly stronger decreased signal intensity in the contralateral ventricles of the group of younger mice. As no significant difference was observed in the volume of the contralateral ventricles (Figure 2B) between the 2 groups, we concluded that a decrease in the rate of CSF turnover was responsible for the reduced contrast agent clearance observed in the 12-month-old mice.

Our previous studies have shown that lymphatic outflow from CSF is reduced in 18-month-old mice compared with 2-month-old mice following a bolus injection of a macromolecular fluorescently labeled tracer (7). We tested whether this difference would be apparent by MRI in 12-month-old mice by quantifying the signal intensity of the contrast agent in regions of interest (ROIs) positioned in the deep and superficial cervical lymph nodes. Dynamic CE-MRI quantification revealed less contrast agent signal in both groups of cervical lymph nodes in the 12-month-old mice compared with the group of younger mice (Figure 2, C and D; and Supplemental Figure 1, B and C). Quantifications of the volume of 3D reconstructions of the cervical lymph nodes also did not show significant differences between the 2 age groups (Supplemental Figure 1, D and E).

In sum, we observed that with aging, a low-rate infusion of a macromolecular contrast agent was associated with reduced clearance from the ventricles and a delayed transport to draining lymph nodes, which is consistent with previous studies by our group and others. Thus, we next aimed to exploit these differences in CSF turnover dynamics between the 2 age groups of mice to help identify major efflux routes from the ventricular system to the lymphatic system.

### Contrast agent flows along the ventral aspect of the brain and down the spine.

**Figure 3:**
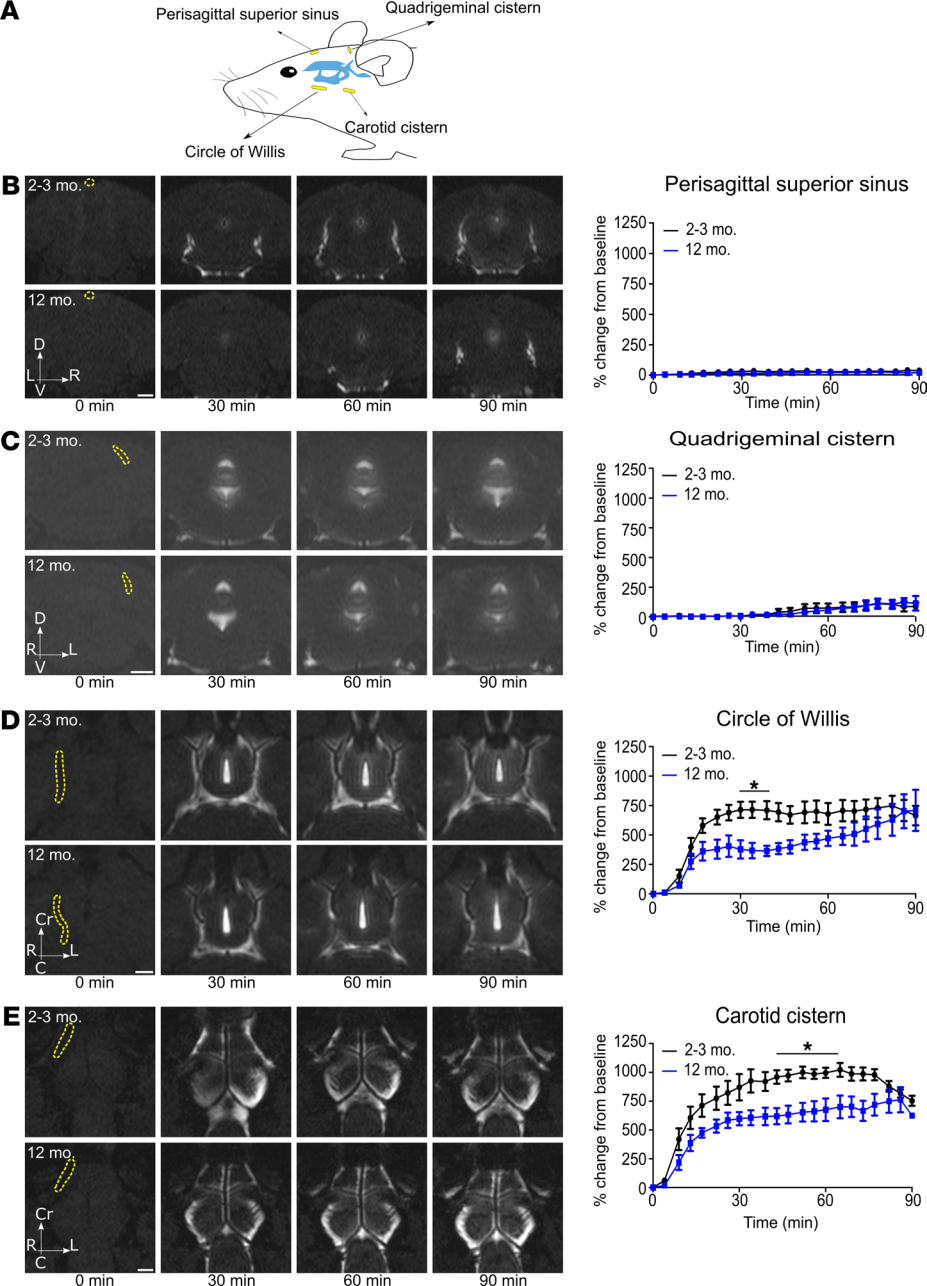
CSF predominantly clears along the ventral aspect of the skull. Visualization of tracer clearance after low-rate intraventricular infusion (0.1 μL/min) of GadoSpin D solution at 25 mM Gd. Data were acquired with a series of T1-weighted MRI measurements (3D time-of-flight gradient recalled echo sequence). (**A**) Overview scheme of ROI location. (**B** and **C**) Coronal sections demonstrating in 2- to 3-month- and 12-month-old mice the dynamics of CSF efflux in representative ROIs (shown in yellow) of the dorsal aspect of the skull: in the perisagittal superior sinus and the quadrigeminal cisterns. (**D** and **E**) Horizontal sections showing the dynamics of CSF efflux in the ventral aspect of the skull in 2- to 3-month- and 12-month-old mice: around the circle of Willis and around the internal carotid (ROIs in yellow). Quantifications of the different ROIs are expressed as the mean ± SEM of 2- to 3-month-old mice (*n* = 7) vs. 12-month-old mice (*n* = 6) and are representative of 3 independent experiments. *P < 0.05 (2-way ANOVA followed by Bonferroni’s post hoc test). Scale bars: 1 mm.

CSF has recently been proposed to reach the dura mater on the dorsal aspect of the skull, where it is hypothesized to be either directly or indirectly drained by meningeal lymphatic vessels leading to the deep cervical lymph nodes (9, 28–30). Previous work has highlighted lymphatic vessels in the dural tissue surrounding the sagittal and the transverse sinuses to be “hotspots” for uptake from the CSF. Thus, following intraventricular infusion of GadoSpin D, we quantified the signal intensity in 2 ROIs of the dorsal aspect of the skull in proximity to these regions. In both areas, we could observe only a limited maximum change in signal intensity compared to baseline (sagittal sinus ROI: <42%; quadrigeminal cistern ROI: <120%) and no significant differences at any time point between the groups of 2-month-old and 12-month-old mice (Figure 3, A–C; and Supplemental Figure 2, A and B).

On the other hand, quantifications of ROIs in the ventral region (at the basal cisterns around the circle of Willis and the internal carotid artery) showed a substantial increase in maximum change in signal intensity compared with baseline (circle of Willis ROI: >700%; internal carotid ROI: >700%), suggesting that this area is a major site of contrast agent bulk flow (Figure 3, A, D, and E; and Supplemental Figure 2, C and D). Moreover, in the 2 regions investigated, the signal intensity was quantitatively significantly higher at earlier time points in young mice compared with the group of 12-month-old mice. These results indicate that an infused macromolecular tracer principally flows with the CSF through the ventral, rather than dorsal, aspect of the skull.

Because CSF leaving the ventricles has free communication with the subarachnoid space around the spinal cord, we also quantified the dynamics in the ventral aspect of the cervical spine (Supplemental Figure 3A). We observed a rapid increase in signal intensity, in the group of young mice, that reached a maximum percentage change of more than 600%. Conversely, in the group of 12-month-old mice, the signal intensity increase was delayed and only reached approximately half of the value observed in young mice. Thus, CSF tracer flow from the ventricular system to the spine can be easily demonstrated with CE-MRI and exhibits the expected decline with aging.

Recent work has introduced the concept of glymphatic flow that would theoretically aid in CSF/solute penetration into the brain parenchyma along para-arterial spaces. Recent MRI studies have shown that low–molecular weight contrast agents, such as gadoteric acid, do indeed demonstrate signal enhancement within the parenchyma (31–36). However, we were unable to confirm a significant influx of the GadoSpin D contrast agent (17 kDa) into the brain cortex in either group (maximum percentage change of 17%) (Supplemental Figure 3B). This limited signal enhancement of a macromolecular contrast agent within the brain is consistent with our earlier study (8) and others (12).

Thus, we conclude from this study that bulk flow routes to the basal cisterns and the subarachnoid space of the spinal canal are major pathways for CSF macromolecular distribution from the ventricular system.

### Dynamics of CSF contrast agent outflow support route(s) leading to the nasopharyngeal lymphatics.

**Figure 4:**
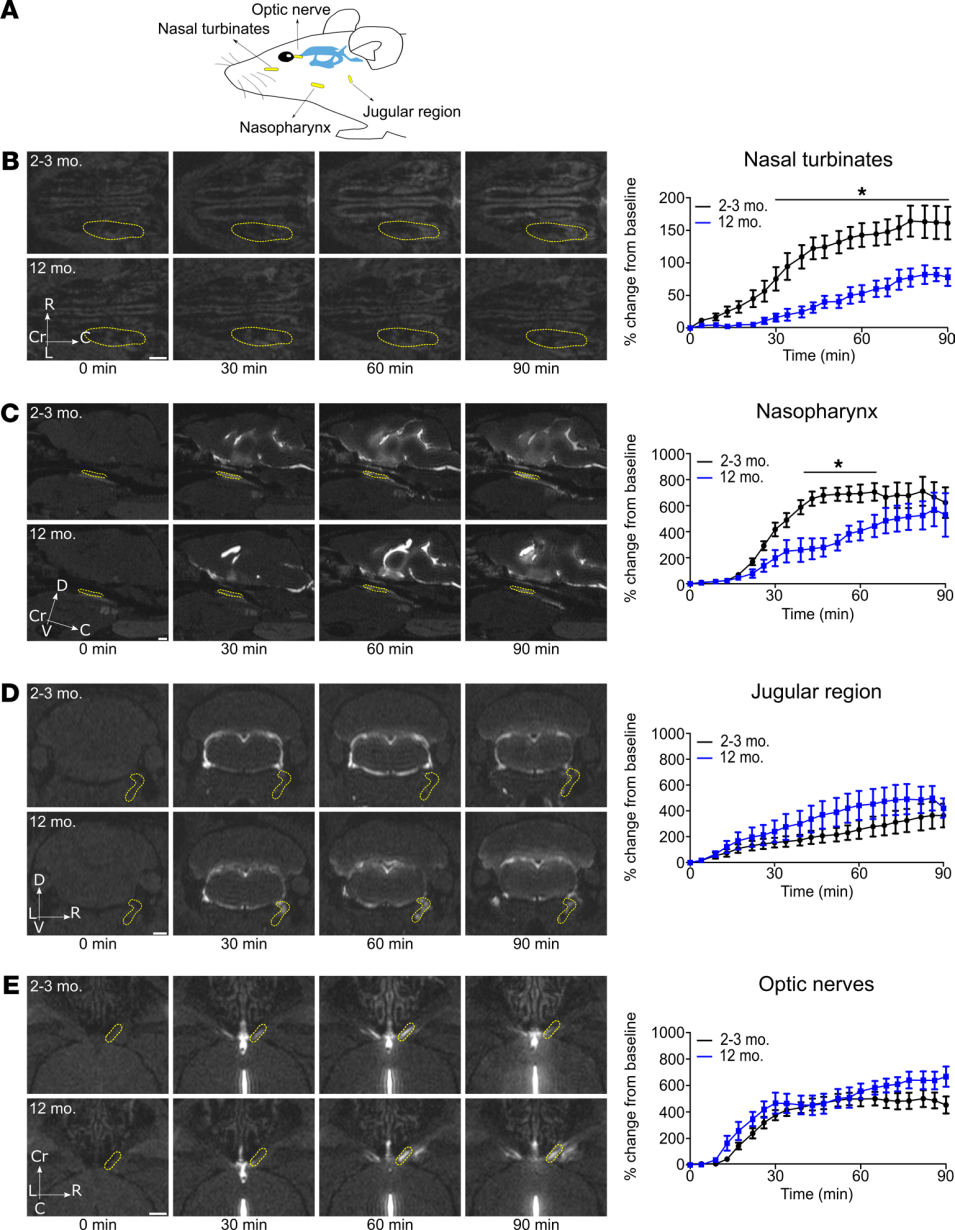
Clearance of CSF from the cranium is reduced with aging in the nasal turbinates and the nasopharynx but not in the jugular region and around the optic nerves. Imaging of tracer clearance after low-rate intraventricular infusion (0.1 μL/min) of GadoSpin D solution at 25 mM Gd. Data were acquired with a series of T1-weighted MRI measurements (3D time-of-flight gradient recalled echo sequence). (**A**) Overview scheme of ROI location. (**B**) Horizontal sections demonstrating the dynamics of CSF efflux to nasal turbinates in 2- to 3-month- and 12-month-old mice. (**C**) Sagittal sections reveal the dynamics of contrast agent in the nasopharynx in the 2 groups of mice. (**D**) Coronal sections demonstrating the CSF efflux from the jugular region in the groups of mice of different ages. (**E**) Horizontal sections showing CSF efflux along the optic nerves in the groups of mice of different ages. Quantifications of the different ROIs (shown in yellow) are expressed as the mean ± SEM of 2- to 3-month-old mice (*n* = 7) vs. 12-month-old mice (*n* = 6) and are representative of 3 independent experiments. *P < 0.05 (2-way ANOVA followed by Bonferroni’s post hoc test). Scale bars: 1 mm.

After observing that a substantial portion of the tracer reached the basal cisterns, we aimed to elucidate the potential anatomical pathways from this location that might be used to reach the cervical draining lymph nodes. Based on our observation that the bulk of the contrast agent appeared to flow continuously from the region of the olfactory bulbs to the nasopharynx and to the draining lymph nodes, we first quantified the changes in signal intensity in ROIs localized in the nasal turbinates and the nasopharynx (Figure 4, A–C; and Supplemental Figure 4, A and B). Dynamic imaging showed, in the group of 12-month-old mice, a delayed and significantly reduced transport of contrast agent in these 2 regions.

At other regions suggested to be efflux sites from the skull, CSF contrast agent signal could be detected in the jugular region below the skull and around the optic nerves (Figure 4, A, D, and E; and Supplemental Figure 4, C and D). However, at these 2 regions, our quantifications revealed that no significant differences in the signal intensity at any time point were observed between the young and 12-month-old groups. In fact, the jugular region appeared to show a trend toward increased signal intensity over time in the 12-month-old mice compared with the young mice, indicating that this region may be a site of accumulation of contrast agent. An earlier report has shown that dural lymphatic vessels of this region become more hyperplastic with age (11).

Thus, because the expected differences in signal dynamics between young and older animals were only detectable in the nasal turbinates and the nasopharyngeal areas, we conclude that this route is a major pathway for CSF clearance from the cranium to the cervical lymph nodes.

### Histological confirmation of CSF outflow route(s) to the nasopharyngeal lymphatics.

Interestingly, the signal quantifications revealed that only a minimal increase of signal (170% in young mice, 85% in 12-month-old mice) could be detected within the turbinates themselves in either group, whereas much larger signal increases were detected at the nasopharynx (710% in young mice, 570% in 12-month-old mice). This may suggest that the contrast agent distributes to a large area throughout the nasal cavity after effluxing through the cribriform plate before draining to the lymphatic vessels near the nasopharynx leading to the lymph nodes, as appears to be evident from the sagittal view in Supplemental Video 1.

**Figure 5:**
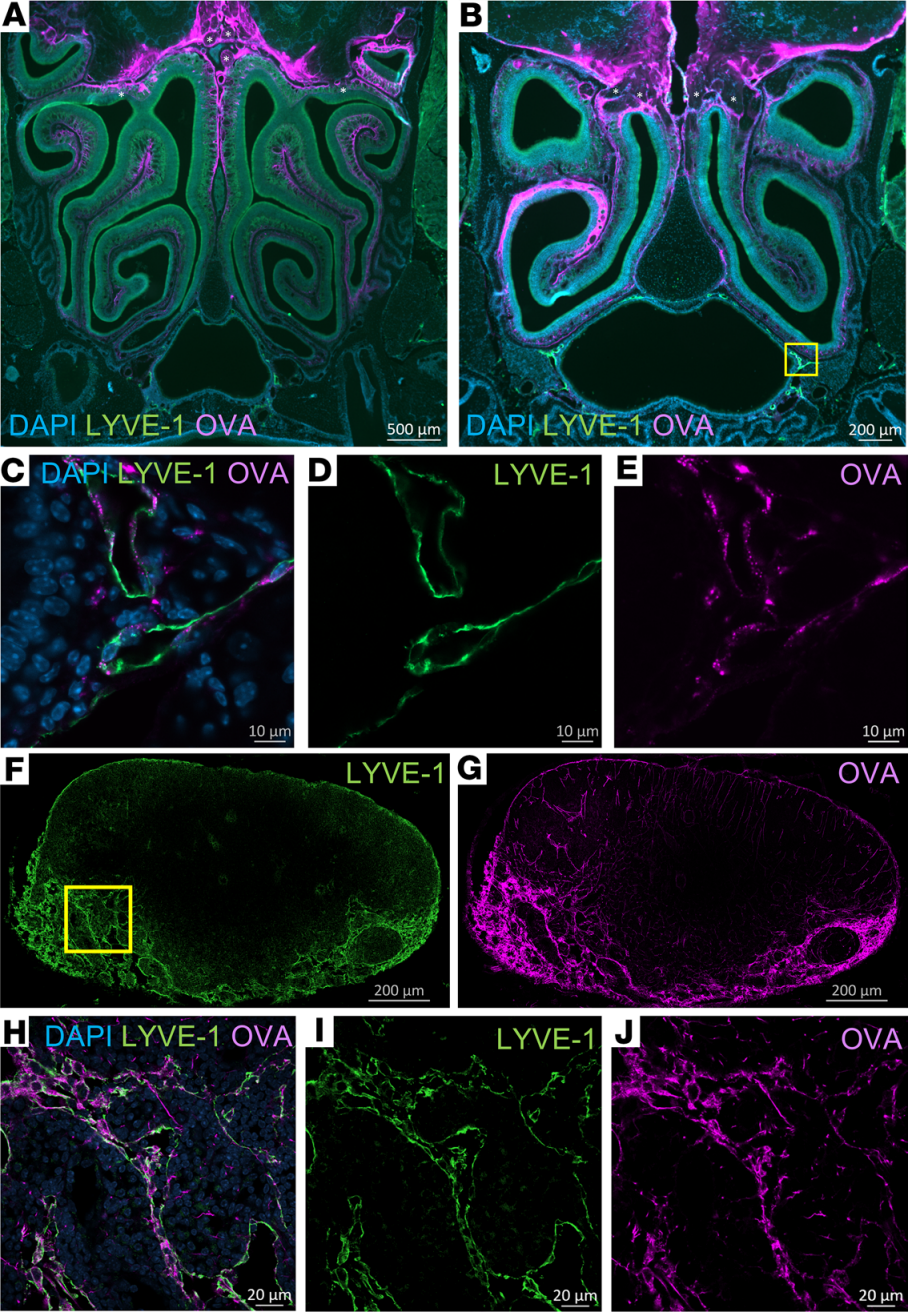
Histological validation of CSF tracer efflux through the cribriform plate to nasopharyngeal lymphatics and to deep cervical lymph nodes. Fluorescently labeled ovalbumin (OVA) was introduced via low-rate intraventricular infusion (0.1 μL/min for 60 minutes), and at 90 minutes the mice were sacrificed for postmortem analysis of tracer efflux. (**A** and **B**) Coronal sections of decalcified skulls demonstrating substantial efflux of OVA into the nasal mucosal tissues. OVA (purple) can be seen crossing the cribriform plate alongside several olfactory nerve bundles (indicated with *). Lymphatic vessels (stained with LYVE-1 in green) can be found in proximity to the nasopharynx under the nasal turbinates. Scale bars: 500 μm (**A**), 200 μm (**B**). (**C**–**E**) High-magnification view of the nasopharyngeal region indicated by the yellow box in **B**, demonstrating OVA signal within the LYVE-1^+^ lymphatic vessels. Scale bars: 10 μm. (**F** and **G**) Sections of deep cervical lymph nodes indicating close association of OVA signal within lymphatic sinuses stained with LYVE-1. Scale bars: 200 μm. (**H**–**J**) High-magnification view of the region indicated by the yellow box in **F**, demonstrating OVA signal within the LYVE-1^+^ lymphatic sinuses. Scale bars: 20 μm. Data are representative of *n* = 6 mice and 2 independent experiments.

To further evaluate how CSF tracers drain through the cribriform plate to reach lymphatics surrounding the nasopharynx, we injected Alexa Fluor 647–labeled ovalbumin (AF647-OVA) using the same slow infusion protocol employed within the MRI and then assessed the presence of tracer on sections from decalcified craniums and deep cervical lymph nodes. Lymphatic vessels were identified using anti–lymphatic vessel endothelial hyaluronan receptor 1 (anti–LYVE-1) antibodies. As seen in Figure 5, A and B, which were taken from 2 coronal sections at different levels of the nasal cavity, AF647-OVA was clearly associated with olfactory nerves crossing the cribriform plate and was distributed throughout a wide volume of nasal mucosal tissue. A rich network of LYVE-1^+^ lymphatic vessels exists laterally on both sides of the nasopharynx, and these vessels could be found to contain AF647-OVA signal (Figure 5, C–E). Deep cervical lymph nodes located downstream of this site also contained AF647-OVA that was found within LYVE-1^+^ lymphatic sinuses (Figure 5, F–J). Thus, this serves as histological confirmation of the CSF drainage route visible on MRI.

## Discussion

In this study, we have assessed CSF outflow by MRI in 2 age groups (2–3 months and 12 months) of mice by utilizing an indwelling catheter system, allowing low-rate infusion of a macromolecular contrast agent during image acquisitions. Similar to previous reports, we found slower dynamics of CSF circulation in the older cohort of mice compared with the younger mice. Imaging of the contrast agent dynamics in the cranial region revealed that the bulk of the CSF flowed ventrally under the brain through the basal cisterns and exited through the cribriform plate to be collected by lymphatics in the nasopharyngeal region.

Our methods are similar to those in recent publications utilizing MRI for examination of CSF flow in rodents (11, 12, 15). In these studies, the authors infused contrast agents during the MRI acquisition into the cisterna magna through a cannula and examined the efflux routes from the cranial cavity. In our study, we chose to infuse into the ventricles, close to the source of production at the choroid plexuses, which allowed assessment of the clearance from the ventricular system, as well as distribution to and efflux from the subarachnoid space.

We observed minimal contrast agent signal in the dorsal region near the dural sinuses or above the cortical hemispheres. Thus, this is contrary to a concept of a major pathway of CSF outflow at the dorsal dura, as proposed in several recent studies in mice (9, 28, 29) and recently extended to the human situation (37). Instead, the dynamics clearly indicated that the majority of contrast agent traversed the basal cisterns with pathways from the cisterna magna to the subarachnoid space around the circle of Willis. This is consistent with both historical data (1, 38) and other recent MRI studies (8, 11, 12, 15, 24, 33). While it is clear that some portion of CSF does reach the surface of the cortical hemispheres as well as the subarachnoid cisterns located near the dural sinuses (12, 13, 39, 40), the significance and ultimate egress route(s) for this flow remain open questions.

As we anticipated, our data indicate a reduction in CSF outflow in older mice consistent with previous reports (7, 11, 14, 41). We were able to detect delays in 12 month-old animals in CSF clearance and transport at several locations along the CSF flow pathways, including the lateral ventricle, basal cisterns, cervical spinal subarachnoid space, nasal turbinates, nasopharyngeal lymphatics, and CNS-draining lymph nodes. Thus, the data are indicative of an overall reduction in CSF turnover with aging. The reason for this diminished turnover of CSF is not yet clear; however, it may be related to a reduced CSF production by the choroid plexuses (27, 42), morphological changes in the dural lymphatics (11), or a reduced transport within lymphatic vessels outside the CNS (43–45). Whether this slowed CSF circulation plays a role in the development of neurodegenerative disorders associated with aging, as speculated in many recent studies (11, 46), remains to be determined.

We took advantage of the differences in contrast agent dynamics between the 2 age groups of mice to attempt to elucidate the relative importance of several potential efflux routes. We hypothesized that only along major bulk flow pathways for CSF egress would the patterns of contrast agent dynamics between the 2 age groups mirror those seen at the downstream lymph nodes. Since we found that limited contrast agent signal was apparent along the dorsal aspect of the skull, we focused these efforts on potential efflux routes from the basal cisterns. From this location, evidence exists in the literature for outflow to the lymphatic system through the cribriform plate (1, 5, 7, 16, 17, 19, 47), along the sheaths surrounding the optic nerves (7, 48, 49), through the jugular foramina (7, 10, 11, 15, 47), and from the spinal column (13, 23, 50). Of these routes, in our study, only the spinal and nasal regions appeared to exhibit the expected contrast enhancement dynamics between the 2 age groups of mice. An outflow pathway to the lymphatic system does indeed exist in rodents from the sacral region of the spine; however, our previous work and others have determined that under normal conditions the spinal pathways are minor compared with the cranial efflux routes (13, 51, 52). Thus, the resulting conclusion of a major CSF outflow pathway through the cribriform plate would be in agreement with many previous studies (14, 18, 53, 54). Strong supporting evidence for this conclusion comes from experiments that blocked this pathway, which resulted in dramatic decreases in tracer recovery outside the CNS and increased intracranial pressure during fluid challenge (53, 55). However, we cannot rule out at this point that more direct pathways may exist, extending from the basal skull to reach the nasopharyngeal lymphatics.

This conclusion appears to conflict with that of Ahn et al. in rats (11), who determined using MRI with a macromolecular contrast agent that basal meningeal lymphatic vessels draining through the jugular foramina are the main route for CSF macromolecular uptake and drainage. It must be noted that in their study Ahn et al. did not investigate any potential efflux through the cribriform plate region. However, another possible explanation for this discrepancy may be due to different experimental conditions between our study and Ahn et al. In a recent, elegant MRI study by Stanton et al., the authors demonstrated that the choice of anesthesia has a significant effect on the amount of efflux through the cribriform plate, with mice under isoflurane anesthesia demonstrating much less gadolinium diethylenetriaminepentaacetic acid signal in this area (15). This study is consistent with earlier work demonstrating differences in CSF flow dynamics under different types of anesthesia (8, 13, 56). The authors demonstrated that mice under 1.5% anesthesia exhibited more signal at the jugular foramina along the cranial nerves and also within the spinal canal, indicating that shunting of the CSF flow may occur under certain conditions (15). This potential redirection of flow is an important factor to consider, especially in the context of pathological conditions such as hydrocephalus or glioblastoma, which may block CSF outflow pathways at the skull and reroute flow to the spine (57, 58).

The situation in humans remains unresolved (6). Studies have presented evidence both for and against efflux of contrast agent through the cribriform plate (54, 59–61). One recent clinical MRI study concluded that CSF efflux to the nasal region is minimal in humans (61). This study used a low–molecular weight gadolinium contrast agent injected into the lumbar intrathecal space and examined the nasal turbinates at multiple time points in patients with various CSF circulation disorders. Although contrast agent was observable in almost half of the patients below the cribriform plate along the olfactory nerves, the authors were unable to observe a significant increase of signal within the nasal cavity at any time point. Our current study demonstrates the technical difficulty of detecting significant contrast agent signal enhancement within nasal tissue using an MRI approach, even though we employed a macromolecular contrast agent that should clear exclusively from the nasal submucosa through lymphatics (53). While the exact anatomical routes remain to be elucidated, it was evident from our decalcified sections after ovalbumin infusion that the tracer spread throughout a wide volume of the nasal tissue after crossing the cribriform plate, which may partially account for the difficulty in detecting signal with MRI in humans.

In sum, through establishment of a technique allowing dynamic CE-MRI under low-rate infusion of gadolinium contrast agents, we conclude that CSF distributes from the ventricles to the subarachnoid space ventral to the brain and in a caudal direction down the spine. Under our experimental conditions, a major outflow route from the cranium appeared to be through the nasal region to reach lymphatic vessels near the nasopharynx before draining to the cervical lymph nodes. With aging, the dynamics of clearance from the ventricles and flow through the nasal turbinates and nasopharyngeal lymphatics to the lymph nodes were reduced. These experiments have set the stage for further MRI evaluation of CSF outflow in mouse models of neurological disorders.

## Methods

### Mice.

Female wild-type mice (Janvier) on a C57BL/6 background were kept under specific pathogen–free conditions until they were used for experimental studies.

### Surgical preparation.

Mice were anesthetized by intraperitoneal injection of 100 mg/kg ketamine and 20 mg/kg xylazine and fixed in a stereotaxic frame (Kopf Instruments). Under this anesthesia, the skull was thinned with a Proxxon GG 12 engraving drill. A 28 G, 2.5 mm long, MRI-compatible microcannula (328OP/PK/Spc; Plastics One) was inserted stereotactically 0.95 mm lateral and 0.22 mm caudal to the bregma and 2.50 mm ventral to the skull surface (13). The microcannula was sealed with cyanoacrylate glue. Animals were transferred into the magnet in a prone position on an MRI cradle (BioSpec Avance III 94/20; Bruker BioSpin GmbH). A 1 to 1.5 m long polyethylene catheter filled with a GadoSpin D (nanoPET Pharma GmbH) solution at a Gd concentration of 25 mM was connected to the MRI-compatible microcannula and a 10 μL syringe operated by an MRI-compatible NanoJet syringe pump (Chemyx Inc.). The skin incision was then closed with a medical adhesive bandage around the cannula and the catheter. Animals were allowed to breathe spontaneously during the entire experimental procedure. Respiratory rate and temperature were measured with noninvasive probes (SA Instruments). Throughout the experiment, the body temperature was maintained between 36.5°C and 37.5°C. During the MRI measurement, the initial anesthetic was supplemented as necessary with 0.5%–1% isoflurane delivered in 98% O_2_ to keep the breathing rate less than 140 breaths/min.

### Dynamic CE-MRI of the head and neck.

Animals were examined in a horizontal-bore 9.4 T animal scanner (BioSpec Avance III 94/20) with a BGA12S gradient system with ParaVision 6.0.1 and a linearly polarized coil with an inner diameter of 40 mm (all from Bruker BioSpin GmbH). Contrast-enhanced imaging was achieved with a 3D time-of-flight gradient recalled echo sequence originally adapted for imaging of peripheral lymph vessels (62) with a recovery time of 12.0 ms, echo time of 2.5 ms, flip angle of 25°, matrix of 600 × 432 × 180, field of view of 36.00 mm × 25.92 mm × 18.00 mm, 1 average, and a scan time of 4 minutes, 19 seconds, 200 milliseconds. A phantom placed in the vicinity of the animal’s head (solution diluted in 0.9% NaCl at 5 mM Gd) was used for image intensity normalization over the time series. Following a precontrast scan, a GadoSpin D solution at 25 mM Gd was infused at a constant rate of 0.1 μL/min for 60 minutes. Total scan time was between 95 and 99 minutes.

### Data processing.

ROIs were manually drawn around the different anatomical regions investigated with Horos (version 3.3.6, Horos Project). Signal intensity was normalized using the reference phantom. The normalized ROI value (provided in Supplemental Figures 1–4) was calculated by dividing the original ROI value by the phantom value of the same scan. Contrast agent efflux over time was determined by calculating the percentage change of signal intensity as a function of time after infusion of the contrast agent using the following equation: [(normalized signal intensity – normalized precontrast intensity)/(normalized precontrast intensity)] × 100. Ventricle and lymph node volumes were quantified using semiautomatic segmentation tools in 3DSlicer, version 4.11 (https://www.slicer.org). All the Digital Imaging and Communications in Medicine (DICOM) image files were imported into 3DSlicer for segmentation and 3D modeling. The ROI was first defined and segmented with the segment editor module. The model maker module was used to create the 3D model. Finally, the volume was determined via the segment statistics module.

### Histological analysis of tracer efflux to lymphatics.

For experiments where ventricular infusions were followed by histological analysis, an identical procedure was used with the following modifications: a solution of AF647-OVA (Thermo Fisher Scientific) dissolved in artificial CSF (Harvard Apparatus) at a concentration of 5 mg/mL was infused at a constant rate of 0.1 μL/min for 60 minutes; the 2- to 3-month-old wild-type mice were sacrificed at the end of the infusion.

For decalcification, intracardiac perfusion with PBS and 4% paraformaldehyde (PFA) was performed. Afterward, the mice were decapitated followed by the removal of skin, muscles, incisors, and the lower jaw from the cranium. The cranium was then immersed in 4% PFA for overnight fixation before being placed in 14% EDTA for 7 days (refreshed daily) at 4°C. Decalcified tissue was then immersed in 30% sucrose for 3 days for cryoprotection before OCT embedding. Then, 20 μm thick coronal sections were cut from a cryostat (CryoStar NX50, Epredia) and stored at –80°C. For immunofluorescence staining of LYVE-1, frozen tissues were first hydrated with PBS for 10 minutes, then permeabilized by 0.1% Triton X-100 for 10 minutes. For blocking, 10% goat serum was used for 1 hour at room temperature. Tissues were incubated with primary antibody (rabbit anti–LYVE-1, AngioBio, catalog 11-034, 1:600 dilution) for 3 hours at room temperature and then washed with PBS before incubating with secondary antibody (goat anti-rabbit Alexa Fluor 488, 1:500 dilution, catalog A27034, Invitrogen) for 2 hours at room temperature. Imaging of the nasal region was done under a Zeiss Axio Zoom.V16 microscope equipped with a Teledyne Photometrics Prime BSI Scientific CMOS camera combined with a CoolLED pE-4000 illumination system and ZEN 2 software (Zeiss). Higher magnification images were then acquired using a Zeiss LSM800 confocal microscope.

For processing of draining lymph nodes, cervical lymph nodes were postfixed in 4% PFA at 4°C overnight. Lymph nodes were further immersed in 30% sucrose for 2 days at 4°C before being snap-frozen in melting isopentane with liquid nitrogen. The frozen tissue was cut serially into 15 μm sections with a cryostat microtome (Leica Microsystems). Sections were incubated with an anti–LYVE-1 (eBioscience, clone ALY7, 1:200) primary antibody for 2 hours at room temperature before incubation with a donkey anti-rat (catalog A21208, Invitrogen, 1:1000) secondary antibody conjugated with Alexa Fluor 488 for 1 hour at room temperature. Sections were finally counterstained with DAPI. ROIs were acquired with a Zeiss LSM 880 Axio Observer.

### Statistics.

Statistical analyses were performed with GraphPad Prism 5. Graphs represent mean ± SEM. Means of 2 groups were compared using an unpaired 2-tailed Student’s *t* test. Two-way ANOVAs were used for comparison, with time points being a within-subject factor and age being a between-subject factor, followed by Bonferroni’s post hoc test. A *P* value less than 0.05 was considered statistically significant.

### Study approval.

All mouse experiments were approved by the Landesamt für Gesundheit und Verbraucherschutz, Saarbrücken, Germany (license numbers 31/2018 and 45/2019).

## Author contributions

YD and STP conceived and designed the study. YD performed the MRI experiments. YD, LX, and AS performed the histology experiments. JK, YD, and STP analyzed the data. AM and YD applied for approval of animal experiments. AM maintained the MRI facility. KF made substantial contributions to the conception of the study. YD and STP drafted the manuscript. All authors have approved the final version of the manuscript and have agreed to be accountable for all aspects of the work.

## Supplementary Material

Supplemental data

Supplemental video 1

## Version 1 12/14/2021

In-Press Preview

## Version 2 02/08/2022

Electronic publication
