# Supplementary material for: Magnetic resonance imaging of cerebrospinal fluid outflow after low-rate lateral ventricle infusion in mice
Source: JCI Insight. 2022 Feb 8;7(3):e150881. doi: 10.1172/jci.insight.150881 (PMC8855808; doi:10.1172/jci.insight.150881)
Supplement: Supplemental data [file jciinsight-7-150881-s024.pdf]

<u>Supplemental Data</u>

Supplemental Figures and Legends

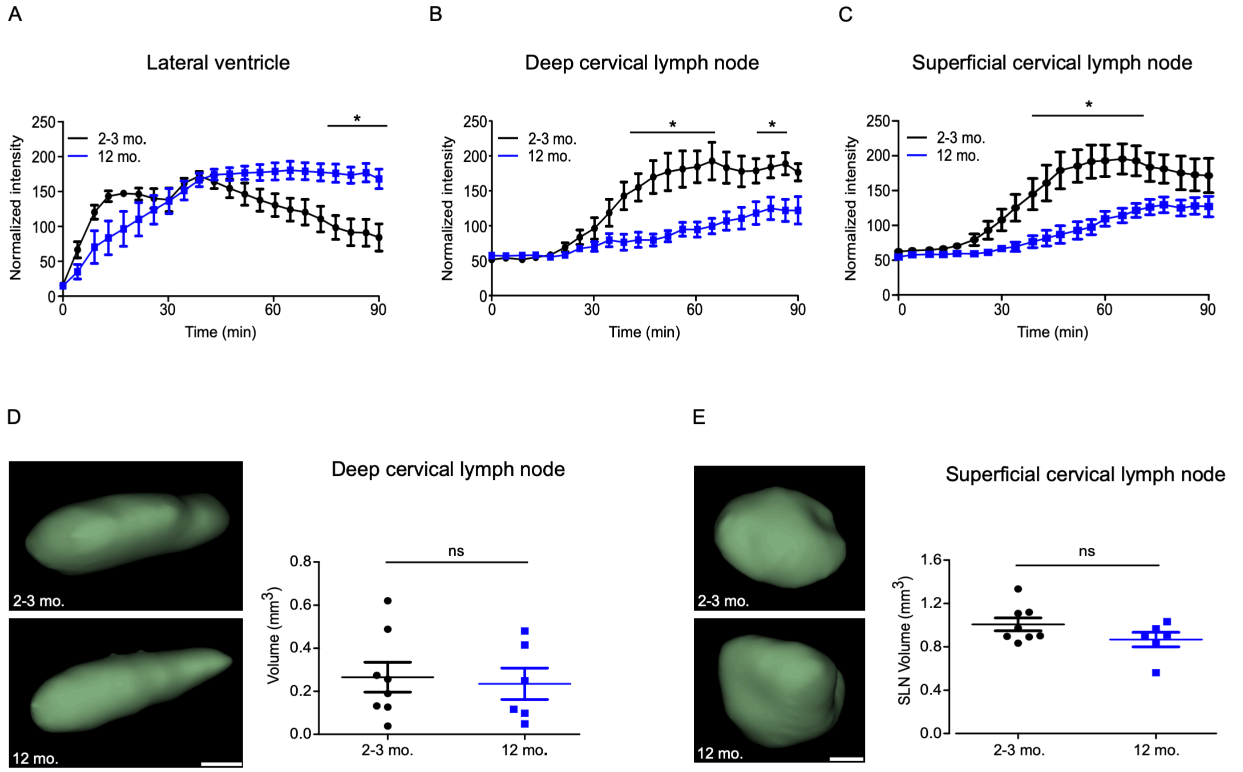

Supplementary Figure 1: Normalized signal intensity values over time of lateral ventricle and cervical lymph nodes of CSF. Signal intensity quantifications were made after low-rate intraventricular infusion (0.1 μL/min) of a Gadospin D solution at 25 mM; data acquired with a series of T1-weighted MRI measurements in contralateral-ventricles (**a**), deep cervical lymph nodes (**b**) and superficial cervical lymph nodes (**c**). Actual signal intensity values were then normalized to a reference phantom. Quantifications of the different ROIs are expressed as the mean ± SEM of n= 7 (2-3 months old mice) vs n=6 (12 months old mice) and are representative of three independent experiments. *p<0.05 (two-way ANOVA followed by Bonferroni's posthoc test). Representative images of 3D reconstruction and volume quantification of deep cervical (**d**) and superficial cervical (**e**) lymph nodes. Lymph node volumes of 2 and 12 month old mice were compared with two-tailed Student's t-test. Scale bars: 0.3 mm.

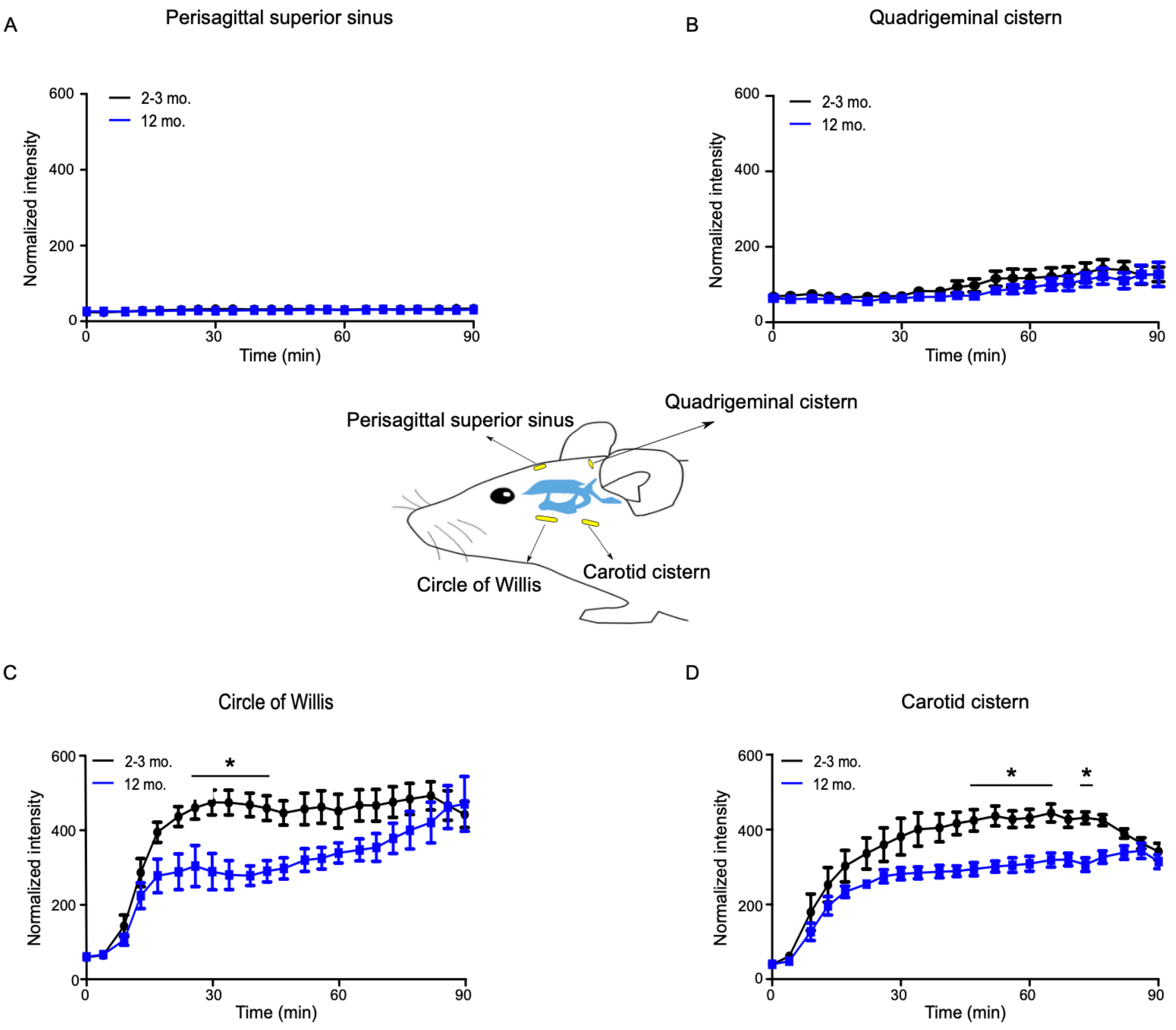

Supplementary Figure 2: Normalized signal intensity values over time in the ventral and dorsal aspects of the skull. Signal intensity quantifications were made after low-rate intraventricular infusion (0.1 μl/min) of a Gadospin D solution at 25 mM; data acquired with a series of T1-weighted MRI measurements at the perisagittal superior sinus (**a**), quadrigeminal cistern (**b**), Circle of Willis (**c**) and internal carotid cistern (**d**). Actual signal intensity values were then normalized to a reference phantom. Quantifications of the different ROIs are expressed as the mean ± SEM of n= 7 (2-3 months old mice) vs n=6 (12 months old mice) and are representative of three independent experiments. *$p<0.05$ (two-way ANOVA followed by Bonferroni's posthoc test).

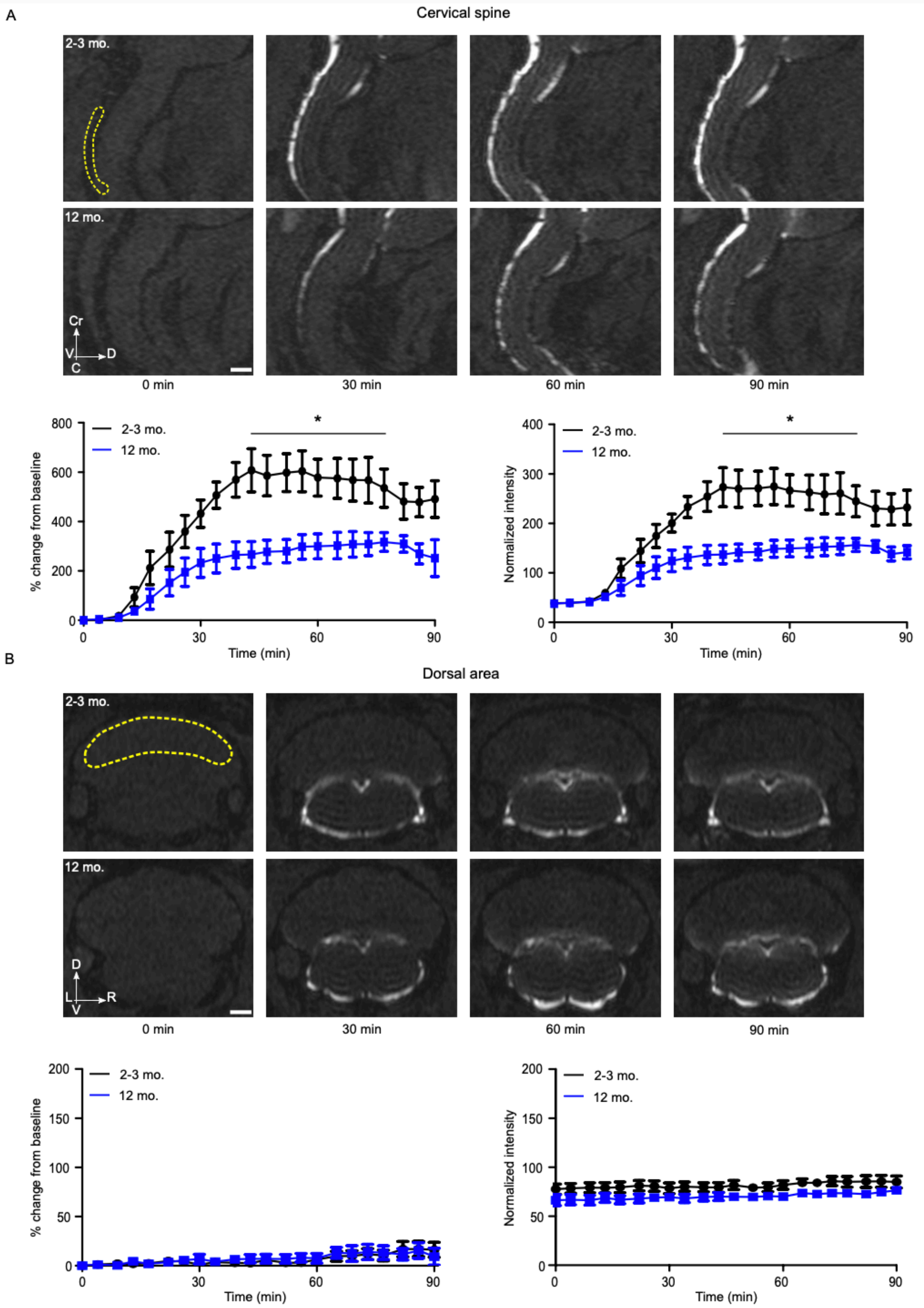

Supplementary Figure 3: Assessment of distribution of CSF tracer to the spinal subarachnoid space and cortical parenchyma. Visualization of tracer spread after low-rate intraventricular infusion (0.1 μl/min) of a Gadospin D solution at 25 mM; data acquired with a series of T1-weighted MRI measurements (3D time-of-flight gradient recalled echo sequence). **a** Signal dynamics of Gadospin D contrast agent at the ventral cervical spine in the sagittal plane in 2-3 months and 12 months old mice. **b** Signal dynamics of the dorsal cortex region in the coronal plane in 2-3 months and 12 months old mice. Quantifications of the different ROIs are expressed as the mean ± SEM of n= 7 2-3 months old mice vs n=6 12 months old mice and representative of three independent experiments. *$p<0.05$ (two-way ANOVA followed by Bonferroni's posthoc test). Scale bars: 1 mm.

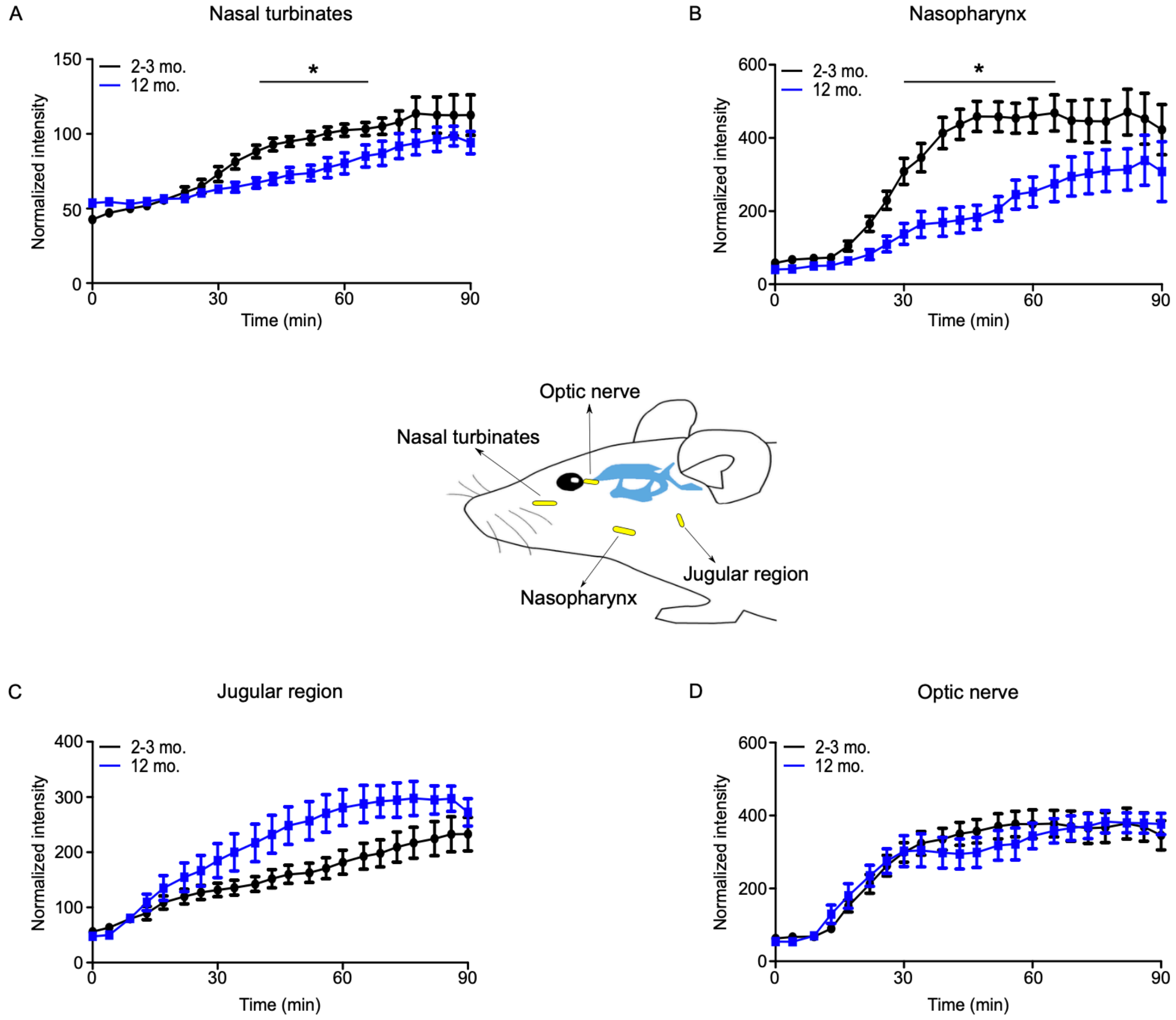

Supplementary Figure 4: Normalized signal intensity values over time of potential CSF outflow sites from the skull. Signal intensity quantifications were made after low-rate intraventricular infusion (0.1 μL/min) of a Gadospin D solution at 25 mM; data acquired with a series of T1-weighted MRI measurements in the nasal turbinates (**a**), nasopharynx (**b**), jugular region (**c**) and optic nerves (**d**). Actual signal intensity values were then normalized to a reference phantom. Quantifications of the different ROIs are expressed as the mean ± SEM of n= 7 (2-3 months old mice) vs n=6 (12 months old mice) and representative of three independent experiments. *$p<0.05$ (two-way ANOVA followed by Bonferroni's posthoc test).

Video Legend

Video 1. MRI of CSF contrast agent efflux from the nasal region through lymphatic vessels to cervical lymph nodes following low-rate ventricular infusion. Maximum-intensity projections video (representative of $n = 7$ mice) showing the spread of tracer after low-rate intraventricular infusion (0.1 μl per min) of a Gadospin D solution at 25 mM gadolinium. Enhancement of the signal intensity in the ventricle is detectable at 4 min, in the nasal cavity at 17 min, and in the neck lymph nodes at 30 min. After 30 min, a continuous signal enhancement from the cribriform plate to the nasopharyngeal lymphatics to cervical lymph nodes is detectable. Images were acquired at 1 frame per 4 min 19 s.
